# Digitally Enabled AI-Interpreted Salivary Ferning–Based Ovulation Prediction: Feasibility Study

**DOI:** 10.2196/73028

**Published:** 2025-08-05

**Authors:** Elizabeth Peebles, William Finlay, Thao-Mi Nguyen, Samuel Barrett, Prudhvi Thirumalaraju, Manoj Kumar Kanakasabapathy, Hemanth Kandula, Carrie Sarcione, Kaitlyn E James, Hadi Shafiee, Shruthi Mahalingaiah

**Affiliations:** 1 Department of Environmental Health Harvard TH Chan School of Public Health Harvard University Boston, MA United States; 2 Department of Obstetrics & Gynecology Massachusetts General Hospital Boston, MA United States; 3 College of Engineering Northeastern University Boston, MA United States; 4 Geisel School of Medicine Dartmouth College Hanover, NH United States; 5 College of Science Northeastern University Boston, MA United States; 6 Department of Medicine Division of Engineering in Medicine Brigham and Women's Hospital Boston, MA United States

**Keywords:** ovulation prediction, salivary ferning, polycystic ovary syndrome, PCOS, irregular menstrual cycles, digital health, reproductive health technology

## Abstract

**Background:**

Females with irregular or unpredictable cycles, including those with polycystic ovary syndrome (PCOS), have limited options for validated at-home ovulation prediction. The majority of over-the-counter ovulation prediction kits use urinary luteinizing hormone (LH) indicators that were optimized for those with regular menstrual cycles exhibiting a predictable mid-cycle LH surge. Artificial intelligence (AI) holds potential to address this health deficit via a smartphone-based salivary ferning ovulation test. Research on populations with irregular menstruation and PCOS can be challenging due to the duration and frequency of menstrual cycles.

**Objective:**

The objective of this study was to evaluate the feasibility for participants with diverse menstrual cycle lengths to complete study tasks designed to train and develop a potential future AI model for salivary ferning–based ovulation prediction.

**Methods:**

Participants were recruited for 2 menstrual cycles where retention, engagement, and adherence were evaluated. Participation entailed remotely collecting and uploading daily data (saliva, LH values), attending lab visits, and returning biological saliva samples.

**Results:**

Of the 133 females recruited from February to October 2023 via targeted patient messages and a public research website, 69 (51.9%) were eligible (age 19-35 years at enrollment, currently menstruating, able to read and comprehend English, weigh more than 110 lb, have an active primary care or gynecological provider, and able to commute to the Massachusetts General Hospital (MGH) main campus within 10 days of their ovulatory event). Of the 43 (62.3%) eligible participants who consented and completed the baseline survey, the majority were White (n=24, 55.8%), employed (n=33, 76.7%), and highly educated (college or more; n=32, 74.4%) and had a mean BMI of 28.9 (SD 7.8) kg/m^2^. Of those who received a study kit (n=29, 42%), 17 (58.6%) participants began data collection, 9 (31%) provided data for completed study tasks for 1 menstrual cycle, and 7 (24.1%) completed the study. Furthermore, 19 (44.2%) eligible participants who completed the baseline survey withdrew from the study, citing menstrual cycles being too irregular for the study timeline (n=5, 26.3%), becoming pregnant (n=4, 21.1%), moving outside the study area (n=4, 21.1%), no time to dedicate to the study (n=2, 10.5%), ineligibility (n=2, 10.5%), and stress related to observing anovulation (n=2, 10.5%).

**Conclusions:**

To optimize future scaled participant completion, the study design would include a more targeted recruitment message to address the high ineligibility status, streamline study procedures to ease the participant burden, and incorporate health education to equip participants with ovulatory health information to ameliorate the potential stress impacts of observing anovulation. After optimization, when scaled, this study design could provide an AI model with sufficient data to develop a smartphone-based ovulation predictor specifically tested on females with irregular or unpredictable cycles, including those with PCOS. A well-informed study design is the foundation to AI advancement and femtech (the technology sector focused on enhancing female health) growth, particularity for ovulatory and fertility digital health.

## Introduction

Femtech, the technology sector focused on enhancing female health, is gaining momentum, particularly with the rise of convenient, at-home, personalized health tracking solutions [1]. Ovulation tracking, through the means of smartphone apps and at-home tests, empowers menstruators with valuable ovulatory and fertility information, allowing them to make informed family planning or pregnancy protection decisions. Additionally, ovulation acts as a vital sign for other health conditions (ie, hormonal, reproductive health) occurring in the body [2]. Most at-home ovulation tests use urinary luteinizing hormone (LH) indicators and are optimized for those with predictable menstrual cycles due to the expected midcycle LH surge. However, menstruators with irregular or unpredictable cycles, including those with polycystic ovary syndrome (PCOS), are often unable to predict ovulation using LH-based tests or receive false-positive results due to tonically elevated or fluctuating LH levels [3].

PCOS, a heterogenous endocrine disorder affecting about 5%-10% of females (up to 21% depending on the criteria and studied population), is characterized by abnormal hormone profiles, disordered ovulation, and irregular menstrual cycles. It is a leading contributor to ovulatory infertility [4-7]. Without a reliable at-home test for ovulation prediction, menstruators with irregular or unpredictable cycles—such as those who are experiencing temporary irregularity (eg, transition off birth control or adjusting to longer cycles) or those with PCOS—face a deficit in options and can experience challenges in understanding their fertility [8].

An accurate at-home ovulation test that does not use LH levels to determine ovulation status would provide those with irregular or unpredictable cycles, including those with PCOS, an opportunity for reliable ovulation prediction. Prior to an LH surge, serum estrogen levels in the body rise and can be detected in multiple biofluids, such as cervical mucus and saliva [9]. In the saliva, changes in estradiol levels correspond with increased concentrations of salivary electrolytes, particularly sodium chloride (NaCl) and certain proteins. The interactions between NaCl and proteins generate consistent fernlike structures due to NaCl crystallization, which we will refer to as *salivary ferning* [10-12]. The concentrations of estrogen, NaCl, and proteins have been observed in a 4-day window leading up to ovulation, corresponding with the 3-5–day fertility window prior to ovulation [9,10,13-15].

Artificial intelligence (AI) has the potential to objectively, accurately, and rapidly analyze salivary-ferning patterns from images captured using an inexpensive smartphone-based optical sensor, helping bridge the ovulatory prediction gap for individuals with irregular or unpredictable cycles, including those with PCOS. AI serves to improve upon the traditional manual ovulation interpretation (ie, at-home tests that require interpretation, such as LH test strips or at-home salivary ferning) by offering a personalized ovulation interpretation that accounts for individual-level fluctuations within the body. Among the various ovulation tests on the market not reliant on LH levels, such as ultrasonography, salivary beta-glucuronidase activity evaluation, serum progesterone, rectal or oral basal body temperature (BBT), and cervical mucus characterization—salivary-ferning analysis offers an at-home, low-cost, and simple method for ovulation assessment [9,16]. BBT is a competitive alternative but, unlike salivary ferning, can be influenced by a multitude of factors, including alcohol consumption, emotional or physical stress, sleep disturbance, change in room temperature, change in waking time, and change in climate [9]. Recent research on salivary-ferning technology performed by this study team revealed that using both artificial and human saliva samples of individuals with regular cycles (n=6), an AI-enabled smartphone device displayed a >99% accuracy in effectively predicting ovulation [16]. The tested saliva did not include irregular or unpredictable cyclers or those with PCOS, leaving this area of research in ovulation development unexplored.

The primary goal of this study was to evaluate the feasibility of remotely collecting saliva samples and LH values over 2 menstrual cycles among menstruators with irregular or unpredictable cycles, including those with PCOS. Specifically, our primary feasibility outcomes were measured by participant recruitment, retention, engagement, and adherence over the study period. Secondarily, we assessed the achievability of recruiting and retaining participants to provide sufficient data to train and develop a future AI model for salivary ferning–based ovulation prediction among regular and irregular menstruators.

## Methods

### Ethical Considerations

The Massachusetts General Brigham Institutional Review Board (IRB) approved this study (approval #2022P001812). During the review of this project, the IRB specifically considered (1) the risks and anticipated benefits, if any, to subjects; (2) the selection of subjects; (3) the procedures for obtaining and documenting informed consent; (4) the safety of subjects; and (5) the privacy of subjects and confidentiality of data. Participants’ nonanonymized data were stored using a Health Insurance Portability and Accountability Act (HIPAA)-compliant program with system, application, and user levels of security. Participants were required to sign e-consent forms to provide informed consent. They were compensated US $50 for each menstrual cycle data provided (2 maximum) and US $75 for completing the conclusion survey, which was sent to all participants who were eligible and had consented.

### Eligibility

To be eligible for the Precision Ovulation Prediction for Patients with PCOS (PEONy) study, a participant must be 19-35 years old at enrollment, currently menstruating, be able to read and comprehend English, weigh more than 110 lb, have an active primary care or gynecological provider, and be able to commute to the Massachusetts General Hospital (MGH) main campus within 10 days of their ovulatory event. The age eligibility criterium aimed to exclude those who could not provide consent for themselves and those experiencing irregular cycles due to perimenopause/menopause [17]. The weight eligibility criterium aimed to exclude those who might be experiencing irregular cycles due to hypothalamic amenorrhea. Participants were excluded if they were currently using hormonal therapy, had a history of surgical menopause (no ovaries or no uterus), had a history of or were currently undergoing chemotherapy or radiation, had a thyroid or prolactin disorder, or were currently breastfeeding or postpartum. These conditions and health statuses can impact cycle regularity [18]. Participants who stopped hormonal therapy were eligible upon having 1 menstrual cycle after cessation (n=1).

### Overall Design

**Figure 1:**
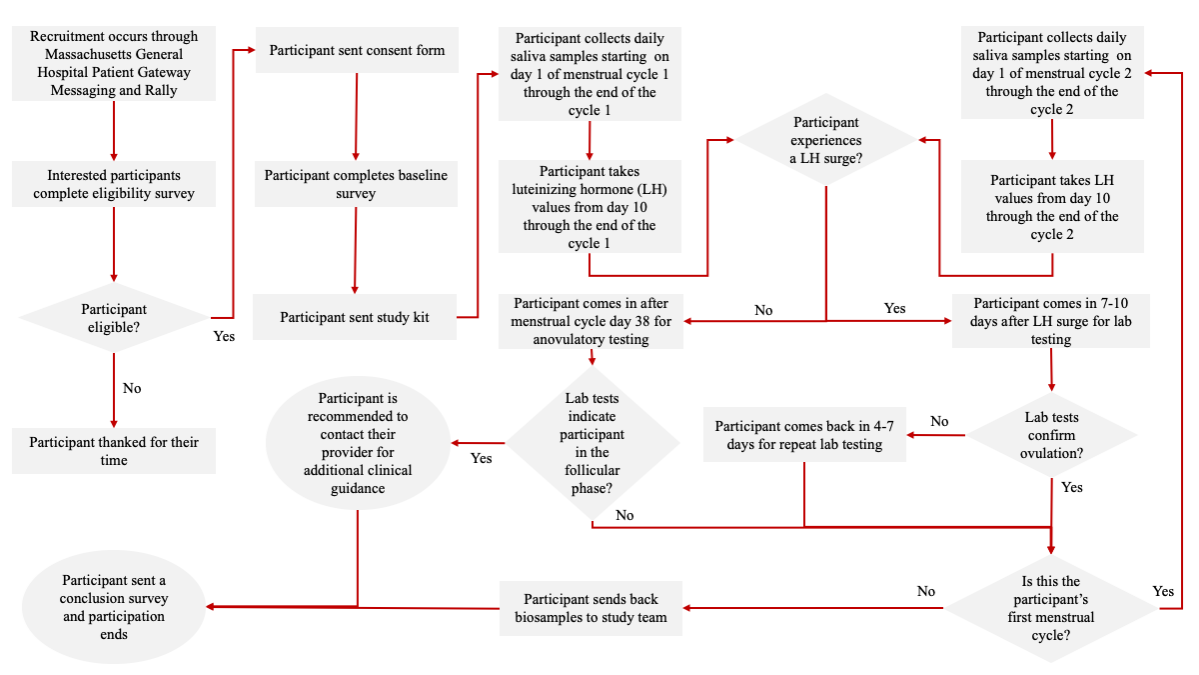
Study summary flowchart of participant tasks from enrollment to study completion.

Once participants were recruited and considered eligible and after they provided consent, they were sent a baseline survey. Upon completing the survey, participants were sent a study kit containing materials to collect and upload daily dried saliva photos and urinary LH values for the duration of 2 menstrual cycles. After an indication of ovulation or after 38 days of data collection, participants commuted to the MGH for lab tests to confirm ovulation. The dried saliva samples were returned to the study team for potential AI model development. Recruitment was defined as participants who engaged with the eligibility survey and consent form. Retention was defined as participants who remained in the study until completion, accounting for those who withdrew or were lost to follow-up. Engagement was defined as the number of required study tasks that were completed. Adherence to the study was determined through the collection of participant self-reported study protocol deviations. These deviations were systematically logged on a study lab notebook and assigned to the specific participant’s profile. A summary of the study tasks from recruitment to study conclusion can be found in Figure 1.

### Recruitment

Participants were recruited into the study from February to October 2023 through 2 methods: the MGH Patient Gateway and the MGH Rally (a public website used for research recruitment). Using the MGH Patient Gateway secure communication, to oversample individuals with irregular cycles, 7130 targeted messages were sent to a group that fell within the target population (*International Classification of Diseases* [ICD] codes of irregular menstruation or PCOS). The message contained the study’s purpose and description, eligibility criteria, participation expectations (time commitment, tasks, travel requirements), compensation, and a link to the study’s MGH Rally post (Multimedia Appendix 1). The MGH Rally post provided the same information as the MGH Patient Gateway message (Multimedia Appendix 2). Interested participants could contact the study email, provided in the MGH Patient Gateway message, or submit their contact information on MGH Rally to trigger study team outreach.

### Informed Consent and Survey Descriptions

Consent forms and participant surveys were distributed via email using the MGH REDCap (Research Electronic Data Capture) system. If an individual expressed interest, they were sent an eligibility survey, and if found eligible, they received an e-consent form. After the consent process, participants received a unique link via email to a baseline survey, which included questions on demographics, reproductive health history, substance use history, and menstrual cycle history. At the end of participation or at the end of the study, whichever came first, all participants who were eligible and had consented were sent a conclusion survey. This survey evaluated reasons individuals chose to participate, obstacles to completing study tasks, study technology, and participant demand, and it offered an opportunity to share additional thoughts.

### Participation

**Figure 2:**
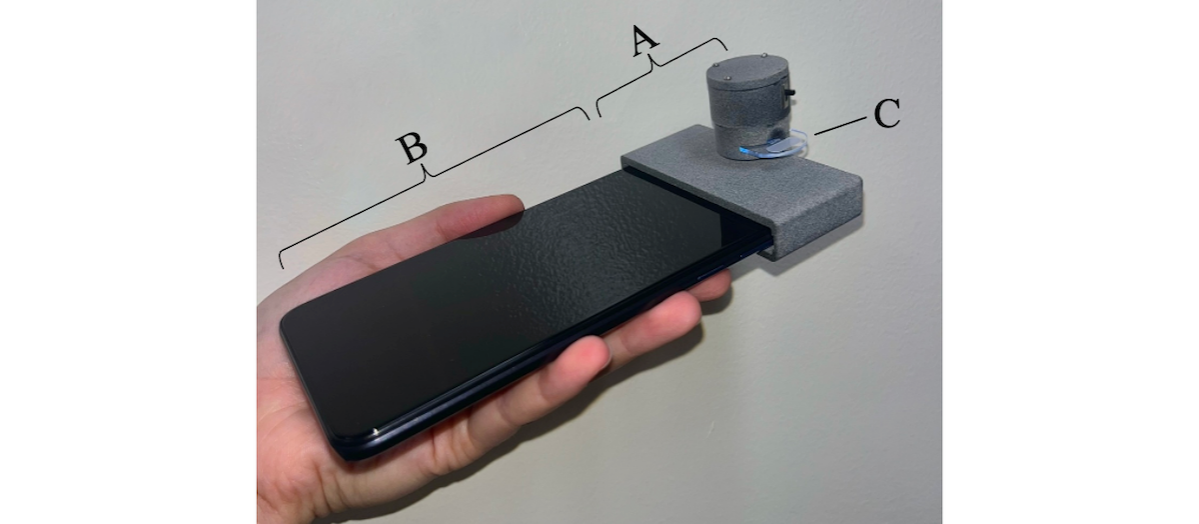
Internally built optical hardware device (A) provided to participants in the study kit that affixes to the study smartphone (B) to assist in capturing illuminated photographs of the dried saliva collection chip (C).

After completing the baseline survey, participants were sent a study kit, which contained a Motorola Moto G Pure phone, an optical hardware device, 100 saliva collection chips, 2 empty chip holders for collected samples, a thin-tip Sharpie, 4 Modern Fertility ovulation prediction kits (80 LH test strips), 4 Modern Fertility pregnancy tests kits (16 pregnancy tests), 2 urine collection cups, 2 lab parking vouchers, a FedEx return shipping label, an instruction manual (Multimedia Appendix 3), and a study calendar (Multimedia Appendix 4). The optical hardware device was built by the study team and made to be attached to the provided smartphone to assist in capturing an illuminated photograph of the saliva collection chips (Figure 2). Each phone contained the predownloaded PEONy app, designed by the study team, which provided instructions and reminders on relevant study actions.

Starting on the first menstrual bleed day of each participant’s cycle, the participant was required to collect daily saliva samples first thing in the morning, before brushing their teeth or consuming food, to exclude discrepancies in the salivary electrolyte concentration. To collect a saliva sample, the participant placed a clean finger under their tongue (bilingual region) and then pressed their finger onto the provided saliva collection chip. The chips were self-labeled with the menstrual cycle number (C1 or C2) and menstrual cycle day (eg, D10) using the provided Sharpie. Once the saliva dried, in approximately 10 minutes, the participant placed the chip in a slot in the optical hardware device provided, affixed the device to align with the front camera of the provided smartphone, and used the PEONy app to upload a photo of the dried saliva. The study team could access the photos once uploaded. On cycle day 10, in addition to collecting and uploading saliva samples, the participants were asked to measure LH values using the Modern Fertility ovulation prediction kits provided and report the value on the PEONy app. Because this was a feasibility study, we did not exclude participants with missing data entries; however, missing data for saliva images and LH values were systematically recorded on REDCap for each participant every day of their cycle(s). Recording missing data will be important for future studies that aim to develop AI models from the saliva images. Once a participant’s reported LH value was greater than 25 IU/L, the participant was scheduled for a lab visit at the MGH main campus within 10 days. This LH trigger value was later redefined to an LH surge (when the LH value rose to a peak and decreased again), which was tracked by the study staff as in some patients, the LH values never surged to 25 IU/L.

The serum progesterone level was obtained at the lab visit to confirm ovulation. After progesterone analysis, the blood sample was disposed of by MGH’s phlebotomy and specimen collection team, following the guidelines administered by the Massachusetts Department of Public Health and the Massachusetts Department of Environmental Protection. Participants continued to record and submit daily saliva samples and LH values until the study team received their lab tests results. If ovulation was confirmed, data collection for 1 cycle was concluded; participants were asked to provide 2 cycles’ worth of data. If ovulation was not confirmed, the participants continued data collection and came in 4-7 days later for another serum progesterone blood test.

If a participant’s LH value did not surge after 38 days, they were instructed to come into the hospital for anovulatory evaluation, including serum pregnancy (beta-human chorionic gonadotropin [bHCG]), estrogen (17B estradiol), and progesterone lab tests. If the lab results indicated that the participant was in the follicular phase, the study team recommended that the participant contact their provider and schedule an appointment for additional clinical guidance.

Participation was considered complete at the return of the labeled saliva collection chips to the study team. Participants who completed the study could be included in potential future AI model training.

### Study Team Communication

To encourage study retention and engagement, ongoing communication was implemented via 2 streams: PEONy app notifications and direct, individual study team communication via email and phone calls. The PEONy app provided reminders at a cadence programmed to study participation tasks (Multimedia Appendix 4). Initially, the study staff communicated upon study kit delivery and after an ovulatory event. As the study progressed and some participants remained inactive over an extended period, communication efforts increased, requiring the hiring of additional study staff. The study staff monitored participants’ tasks each day to track adherence. In the event a participant did not complete study tasks as scheduled, such as not uploading daily data, additional study staff outreach occurred (phone call or email or both) to encourage reengagement in study activities.

## Results

### Recruitment

**Figure 3:**
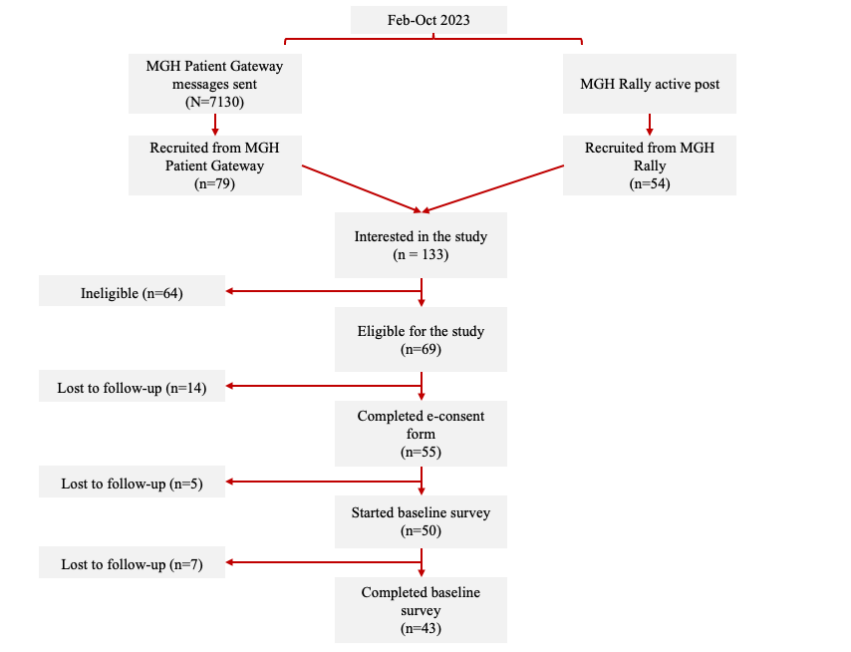
Participant flowchart displaying study recruitment and beginning study tasks. MGH: Massachusetts General Hospital.

From the 7130 MGH Gateway recruitment letters sent out, 79 (1.1%) individuals contacted the study staff via email. An additional 54 individuals expressed study interest through the MGH Rally platform. Slightly over half of the individuals (n=69, 51.9%) who completed the eligible screener were found to be eligible for participation. Criteria limiting eligibility included use of hormonal birth control (n=35, 54.7%), thyroid or prolactin disorder (n=9, 14.1%), not having an active primary care or gynecological provider (n=7, 10.9%), age (n=6, 9.4%), being postpartum (n=6, 9.4%), not currently menstruating (n=4, 6.3%), and weighing less than 110 lb (n=3, 4.7%). (Of note, ineligibility criteria percentages summed to >100% due to individuals being ineligible for multiple criteria.) Of those found to be eligible (n=69, 51.9%), 55 (79.7%) participants completed the consent form, 50 (72.5%) started the baseline survey, and 43 (62.3%) participants completed the baseline survey. A participant flow diagram describing participant retention can be found in Figure 3.

### Data Contribution

**Figure 4:**
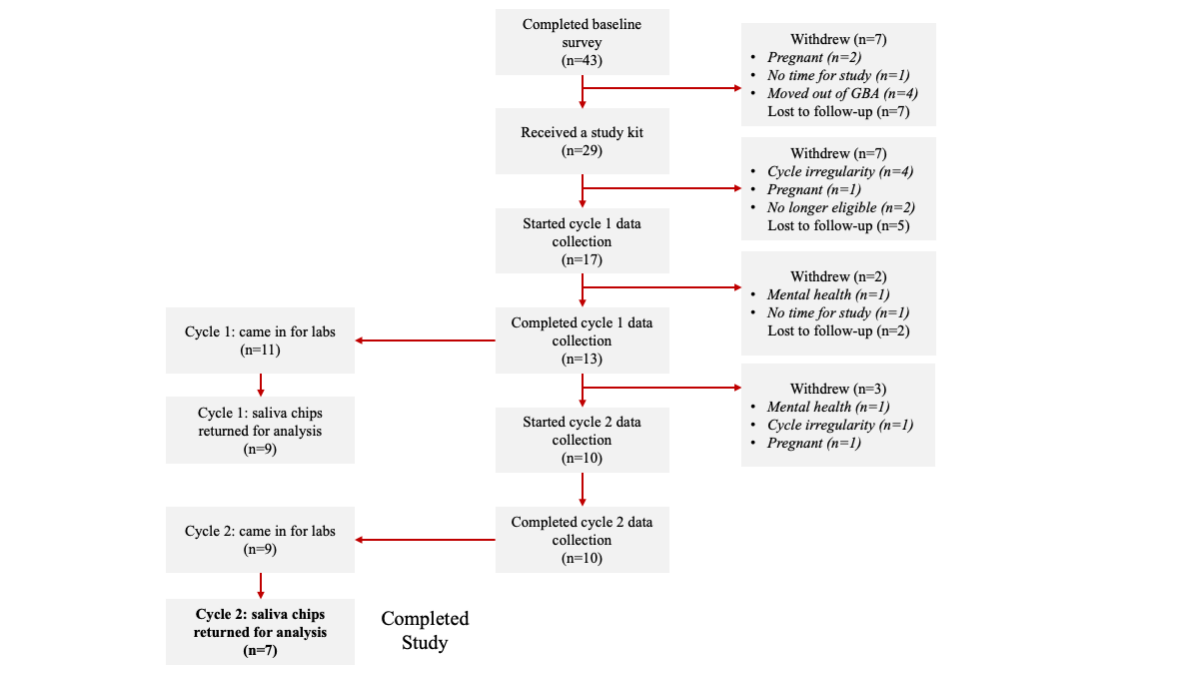
Participant flowchart displaying participant retention and engagement for those who received a study kit. GBA: Greater Boston area.

From February to October 2023, 29 (52.7%) participants received a study kit. The median wait time to receive a study kit after enrolling was 11 (IQR 6-71) days, with a minimum of 1 day and a maximum of 156 days; 11 (37.9%) participants waited over 2 months for a study kit after enrolling. A participant retained the study kit for a median of 112 (IQR 34-151) days, with a minimum of 12 days and a maximum of 418 days, excluding those who never returned the study kit (n=6, 20.7%). In addition, 8 study kits were lost in transit, and 6 study kits were never returned due to participants being lost to follow-up. Ultimately, 23 (79.3%) participants who received a study kit returned it regardless of data contribution. Participant data contribution reflecting participant retention and engagement is summarized in Figure 4. Overall, of those who consented to the study, 7 (12.7%) participants completed the study. The average duration of data contribution for cycles 1 and 2 was 19 and 26 days, respectively. Those who withdrew after starting data collection (n=4, 23.5%) averaged 17 days of data contribution. Regarding protocol adherence, only 1 (5.9%) participant who began data collection reported a deviation (brushing their teeth before their day 1 sample).

### Demographics

Of those who showed interest in the study and were eligible (N=133), demographic data were available for 69 (51.9%) participants, 55 (41.4%) participants provided informed consent, and 43 (32.3%) participants completed the baseline survey. Among participants who provided informed consent (n=55, 41.4%), demographics were further stratified for those who began data collection (n=17, 30.9%) and those who completed the study (n=7, 12.7%), as shown in Table 1.

Of the 43 (62.3%) participants who were eligible, provided consented, and completed the baseline survey, the majority were White (n=24, 55.8%), employed (n=33, 76.7%), and highly educated (college or more; n=32, 74.4%) and had a mean BMI of 28.9 (SD 7.8) kg/m^2^ (Table 1). The majority of participants (n=26, 60.5%) also reported that their health was about the same as that of other people their age. About two-thirds of participants (n=29, 67.4%) reported a PCOS diagnosis, and just over half of the participants (n=15, 34.9%) reported irregular cycles at the time of enrollment (Table 1). The group that completed the study had similar self-reported demographics and health data, but the sample size was too small to make statistical comparisons. Everyone who completed the study (n=7, 100%) had PCOS, and 14.3% (n=1) experienced irregular cycles (<21 days, >35 days) at the time of enrollment (Table 1). More detailed demographic and health information about the 7 participants who completed the study can be found in Multimedia Appendix 5.

**Table 1:** Demographics and health characteristics of those who were enrolled, who consented, and who completed the baseline survey, further stratified by those who began data collection and who completed the study.

Characteristics		Eligible and completed baseline surveys (n=43)	Provided some data (n=17)	Completed the study (n=7)
Age at baseline (years), mean (SD)		28.8 (4.8)	28.4 (4.9)	29.6 (6.4)
**Race/ethnicity, n (%) **				
	White	24 (55.8)	8 (47.1)	3 (42.9)
	Black/African American	3 (7)	1 (5.9)	0
	East Asian	0	0	0
	Southeast Asian	0	0	0
	South Asian	2 (4.7)	0	0
	Middle Eastern	1 (2.3)	1 (5.9)	1 (14.3)
	Native Hawaiian or Other Pacific Islander	0	0	0
	American Indian	0	0	0
	Hispanic	8 (18.6)	5 (29.4)	2 (28.6)
	Other	1 (2.3)	1 (5.9)	0
	Multiple races	4 (9.3)	1 (5.9)	1 (14.3)
**Gender identity, n** **(%)**				
	Female	42 (97.7)	16 (94.1	7 (100)
	Nonbinary/third gender	1 (2.3)	1 (5.9)	0
BMI (kg/m^2^) at baseline, mean (SD)		28.9 (7.8)	29.2 (7.2)	27.9 (6.7)
**Occupational status, n** **(%) **				
	Paid employment	33 (76.7)	13 (76.5)	5 (71.4)
	Unemployed	4 (9.3)	2 (11.8)	2 (28.6)
	Not employed outside of home	2 (4.7)	0	0
	Student	3 (7.0)	2 (11.8)	0
	Retired	0	0	0
	Not working due to disability	1 (2.3)	0	0
**Highest grade or level of schooling completed at the time of enrollment, n** **(%)**				
	Less than 8^th^ grade	0	0	0
	8^th^-11^th^ grade	0	0	0
	12^th^ grade or completed high school	1 (2.3)	0	0
	Post–high school training other than college	2 (4.7)	2 (11.8)	0
	Some college	8 (18.6)	4 (23.5)	1 (14.3)
	College graduate	17 (39.5)	6 (35.3)	2 (28.6)
	Postgraduate (eg, master’s degree, professional, doctorate)	15 (34.9)	5 (29.4)	4 (57.1)
**Smoking status, n (%)**				
	Smoked at least 100 cigarettes in your lifetime	4 (9.3)	1 (5.9)	0
	Ever smoked weed or marijuana in lifetime	26 (60.5)	7 (41.2)	3 (42.9)
**Self-reported overall health, n (%) **				
	Excellent	7 (16.3)	4 (23.5)	1 (14.3)
	Very good	18 (41.9)	7 (41.2)	4 (57.1)
	Good	16 (37.2)	6 (35.3)	2 (28.6)
	Fair	2 (4.7)	0	0
	Poor	0	0	0
**Self-reported health compared to other people of the same age, n (%)**				
	Better than most	12 (27.9)	3 (17.6)	2 (28.6)
	About the same as most	26 (60.5)	13 (76.5)	4 (57.1)
	Worse than most	5 (11.6)	1 (5.9)	1 (14.3)
**Cycle pattern, n (%)**				
	Self-reported regular (21-35 days)	23 (53.5)	11 (64.7)	6 (85.7)
	Self-reported irregularity (<21 days, >35 days)	15 (34.9)	4 (23.5)	1 (14.3)
	Do not know	5 (11.6)	2 (11.8)	0
**Self-reported PCOS^a^ diagnosis status, n (%)**				
	Yes	29 (67.4)	16 (94.1)	7 (100)
	No	14 (32.6)	1 (5.9)	0
Age at PCOS diagnosis (years), mean (SD)		20.4 (5.3)	18.5 (3.8)	17.0 (2.5)

^a^PCOS: polycystic ovary syndrome.

### Study Team Communication

Increased communication efforts occurred 6 months into the study. Additional communication allowed the study team to quickly identify study kits lost in transit, facilitate participation, and reduce the time to withdrawal of those no longer interested in the study. As a result, participant possession of study kits reduced from, on average, 179 days to 93 days. In attempts to recollect study kits, a total of 328 individual outreaches were sent across 28 participants, totaling about 12 outreaches per participant.

### Withdrawn or Lost to Follow-Up

Among participants who provided informed consent and completed the baseline survey (n=43, 62.3%), 36 (83.7%) did not complete the remainder of the study, defined as not accomplishing the following for 2 menstrual cycles: remotely collecting and uploading daily data, attending lab visits, and returning biological saliva samples. Of these, 1 (2.3%) participant never attended lab visits due to study miscommunication, and 2 (4.7%) participants did not return saliva samples (protocol miscommunication: n=1, 50%; lost in transit: n=1, 50%). In addition, 19 (44.2%) participants withdrew; the reasons for withdrawal included menstrual cycles being too irregular for the study timeline (n=5, 26.3%), becoming pregnant (n=4, 21.1%), moving outside the Greater Boston area (GBA; n=4, 21.1%), no time to dedicate to the study (n=2, 10.5%), ineligibility (n=2, 10.5%), and stress related to observing anovulation (n=2, 10.5%). The remaining 40 (58%) participants were lost to follow-up, 7 (17.5%) of whom received study kits and 2 (5%) of whom started data collection (Figures 3 and 4).

### Conclusion Survey

A total of 14 (25.5%) participants completed the conclusion survey, with 10 (71.4%) providing some or all data (with n=9, 90%, completing cycle 1) and 4 (28.6%) contributing no data. Those who did not contribute data withdrew due to their cycles being too irregular to begin participation (n=3, 75%) or because they moved outside of the GBA (n=1, 25%). Of those who took the survey, 7 (50%) had irregular cycles: 4 (57.1%) reported that their irregular cycles made it more difficult to complete the study, while 2 (28.6%) reported that it discouraged them from participating altogether. When asked about the in-person component, 11 (78.6%) respondents indicated they would be more likely to complete the study if the lab testing was located closer to home. Of the 13 (92.9%) participants who received a study kit and instructions, everyone understood the saliva collection protocol. Among the 10 (71.4%) who contributed some data, 8 (80%) respondents reported that the number of tasks was manageable, while 6 (60%) found the daily demand to be challenging, and 1 (10%) respondent mentioned that it was hard to bring the materials when traveling—a common issue reported by participants, resulting in delays in initiating data collection. For urine collection, 5 (35.7%) participants reported that it was or would be inconvenient, with some expressing concerns about sanitation and time commitment. The remaining questions on the conclusion survey asked about study communication, the PEONy app, and the technology used for collecting and uploading data. Regarding study communication, 5 (35.7%) respondents requested more frequent reminders. No participants reported that the app notifications were effective. Multiple participants reported inaccurate programmed time on the phone, making reminders and submissions more difficult. One respondent suggested that notifications should be sent to their personal devices instead of the study kit phone provided, with another participant noting the study would be easier to complete using their personal phone overall. Among the 11 (78.6%) participants who responded to the conclusion survey who opened the PEONy app, 9 (81.8%) reported that it was easy to navigate, and no one reported it being difficult or very difficult to use; however, 7 (70%) of the 10 participants who contributed data did not use any app assistance features. Half (n=5, 50%) of those who collected some data reported technical issues, including app crashing (n=2, 40%) or inaccurate programmed time on the phone (n=3, 60%). There were also some app technical issues for those who did not ovulate and those who did not get periods. Lastly, some respondents wrote that the optical hardware device was flimsy, with 1 (10%) participant reporting that by the end, they were using a personal phone flashlight to improvise.

## Discussion

### Principal Findings

This study was conducted to determine the feasibility of participants with mostly irregular or unpredictable cycles, including individuals with PCOS, to complete the study tasks necessary to generate the longitudinal, image-based data required to train a future AI model for salivary ferning–based ovulation prediction. No AI model was trained or developed as part of this feasibility phase. Of the 133 females with diverse menstrual cycle lengths, recruited from February to October 2023 via targeted patient messages and a public research website, 69 (51.9%) were eligible for the study. Of those who received a study kit (n=29, 42%), 17 (58.6%) participants began data collection, 9 (31%) provided data for completed study tasks for 1 menstrual cycle, and 7 (24.1%) completed the study.

### Comparison With Prior Work

Previous research studies have successfully recruited and retained participants with regular cycles using study designs with a similar [19-21] or a greater [22-24] participant burden (ie, longer time contribution, more daily tasks, more in-person visits) compared to our study. However, menstrual cycle research within populations with irregular or unpredictable menstruation, including those with PCOS, is highly nuanced. The desired study population makes up a small percentage of the total population (25.6%) and is further complicated by the duration and frequency of menstrual cycles and ovulation patterns [25]. Irregular or long menstrual cycles can delay the start date or length of expected daily data contribution, given participants with irregular cycles or PCOS can contribute ~20 days more per cycle than those with regular cycles. For example, a previous ovulation study that collected participant heart rate and BBT recruited fewer participants with irregular cycles compared to participants with regular cycles, and those with irregular cycles contributed fewer cycles per person, in part due to >20% of participants with irregular cycles being unable to determine ovulation days [23]. Additionally, ovulation testing is a sensitive subject due to implications related to health and current or future family planning. Throughout the study, multiple participants withdrew due to stress concerns related to observing anovulation. The study team must keep this in mind when communicating with participants, particularly those in this population who may ovulate infrequently or be anovulatory. Additionally, providing participants with menstrual and ovulation health educational materials upon enrollment could help ameliorate the potential stress related to observed anovulation, especially given low fertility awareness seen in a cross-sectional study of reproductive females across the United States [26]. The health educational material could be delivered through the PEONy app or be printed out to be placed in the study kit. This website pop-up or information sheet would explain the physiology and broader health effects of anovulation. This study and future studies on this population need to account for these nuances, especially in the setting of remote data collection with no in-person study staff support, to create an opportunity to advance female health through at-home, affordable, AI-enabled digital health assays. This smartphone-based salivary ovulation prediction method would provide a reliable opportunity for those with irregular or unpredictable cycles around 35-45 days in length. Those with severe cycle irregularity, such as extreme oligomenorrhea, where ovulation events are highly infrequent (4 or fewer times a year), would need to consult a provider for proper ovulatory care [27].

### Future AI Development

Future large-scale efforts using recruitment and retention techniques learned from this feasibility study can enable the future development of deep learning models for ovulation detection in individuals with irregular cycles. Such a model would build on prior architectures that our study team has used, such as MobileNet (convolutional neural networks [CNNs] implemented on a smartphone-compatible platform), which has previously detected salivary ferning–based ovulation patterns in individuals with regular menstrual cycles with over 99% accuracy [16]. However, new challenges are introduced for individuals with irregular cycles, including increased variability in ferning morphology and inconsistent ovulatory timing, as observed in this feasibility study.

Although ferning-based analyses in those with irregular cycles have not yet been attempted, the previous literature across the broader reproductive health and physiological modeling fields reveals potential areas of adjustments [23,28,29]. For example, Yu et al [23] observed a severe drop in model sensitivity (from ~71% to ~36%) when applying an ovulation prediction algorithm trained on BBT and heart rate data of those with regular cycles to users with irregular cycles. Therefore, adaptations, likely involving a hybridized architecture that combines image classification with robust temporal modeling, may be necessary.

Transfer learning, where models pretrained on larger datasets are fine-tuned on smaller, specific cohorts, may be a viable approach by offering a valuable initialization point, especially in this setting where recruiting and retaining individuals with irregular cycles is challenging [30]. Additionally, synthetic data augmentation with generative models may also help overcome data scarcity and variability [30-32]. In practice, such a strategy could involve model training using a large dataset of ferning patterns and time series information from regular cycles and then adapting or fine-tuning it using data from an individual user with irregular cycles. Combining this with synthetic augmentation of salivary-ferning images, particularly to simulate rare or ambiguous ferning patterns, may help address class imbalance and increase the robustness of classifiers. This type of hybrid approach can help the model recognize broad population-level trends, while still capturing the unique patterns present in an individual’s cycle.

Additionally, advanced time series models, such as those specifically designed to handle uneven sampling or missing data, are likely needed to effectively capture the complex and variable dynamics of ovulation. For example, capitalizing on approaches such as graph neural networks can be used to restructure uneven data into short, aligned segments, making it easier to detect trends even when observations are collected irregularly—a practicality challenge when working long cycle periods [33]. This could help better associate ferning patterns and hormonal data across time, despite inconsistent sampling. Similarly, adversarial learning methods could be another viable approach, where it has been observed to improve forecasting by capturing both overall trends and sharp day-to-day shifts, which could help in predicting ovulation in irregular cycles, where subtle changes can be clinically meaningful but easy to miss using traditional models [34]. In addition to architectural considerations, interpretability will be essential and should remain a central focus throughout model development and implementation [35]. Models that combine CNN-based image analysis with time series prediction should incorporate tools such as saliency maps and attention mechanisms to help trace predictions back to meaningful physiological features and temporal patterns. This transparency is critical not only for clinical validation but also for building user trust and supporting informed, individualized decisions in fertility tracking.

### Strengths and Limitations

This study has many strengths, including successful participant recruitment, retention, engagement, and adherence in collecting menstrual cycle data that could train an AI-model for salivary ferning–based ovulation tracker development. Recruitment efforts engaged 69 eligible participants, more than the study’s target sample size of 50, and MGH Patient Gateway and the MGH Rally post recruitment methods offered an alternative to direct recruitment through clinics, such as infertility clinics, which facilitated the recruitment of a population outside of the infertility treatment setting [36]. The sample size goal of 50 subjects was chosen to allow us to test our technology on those experiencing irregular cycles in a pilot setting [16], as well as measure participant recruitment, retention, engagement, and adherence; proof of concept; and pilot testing of the PEONy app. Second, this study was mostly administered remotely, a strength of the study. This study design was chosen to reduce the participant burden by limiting scheduling and traveling commitments, increase participant accessibility by reaching a more diverse study population (eg, shipping all study materials, including a smartphone), and optimizing the study staff’s time. Although adherence can be harder to track with remote studies, we were able to check whether participants submitted daily data, and we were able to communicate with participants if there was a protocol deviation (ie, sample taken after brushing teeth). Additionally, a future AI salivary ferning–based ovulation predictor would require remote collection, so this study design mimicked real-world application. Retention levels were high among those who started data collection (7/17, 41.2%) relative to the recruited cohort (7/55, 12.7%), given the daily data contribution demand. Increased study communication between the study team and the participants increased participant engagement, particularly when a participant missed a daily data upload. Future work could use the PEONy app to send out custom notifications based on missing data. Additionally, reported deviations regarding data collection, study actions, and technology submission affected a modest percentage of participants. Consistent with previous research, organized and persistent communication, facilitated by hiring additional research staff, contributed to a higher retention and engagement rate [37]. This suggests that funding for menstrual cycle and AI data collection studies would require adequate budgeting for staffing to ensure contact and follow-up.

Though there were many strengths, there were also study challenges, particularly participant ineligibility, attrition, limited study material, time and travel burdens, and technological issues. A large proportion of interested participants (48.1%) were ineligible at screening, with current hormone use being the most common ineligibility criterium. Those with irregular cycles or PCOS commonly manage cycle length and regularity via hormones, which would alter the body’s natural LH and estradiol surges and cause ovarian suppression [38]. The high ineligibility due to hormone use was not surprising, especially given Peters et al’s [19] experience with similarly high levels of ineligibility at screening, even for participants with regular cycles. However, a more targeted recruitment method that addresses this ineligibility criterium should be implemented in future studies using the target population of those with irregular cycles. Additionally, recruited participants were still medically interfaced (required medical provider or contacted through hospital-affiliated email addresses), and the study required intensive participant involvement and frequent interaction with technology, which may limit generalizability. Furthermore, additional limitations on generalizability include eligibility requirements of English literacy, travel ability, and access to health care; however, ferning biology is likely not to be affected by these. Second, only 7 participants completed the study, and these participants were slightly older, with higher education levels than the cohort that consented and completed the baseline surveys, although significant comparisons are across small cohorts. A larger, more diverse sample may increase generalizability. Limited and lost-in-transit study materials resulted in delays in participants receiving study kits, with 37.9% of participants waiting over 2 months to receive one. Confirming the participants’ home address and mandating the participants to sign for the package helped minimize lost-in-transit study materials. Participants were also subject to rapid reproductive and geographic status changes. The ability to collect data using the study kits immediately upon enrolling and simultaneously across participants necessitates sufficient supplies. Furthermore, using outpatient lab services closer to participants’ home address could prove instrumental in increasing recruitment and retention. This adjustment in design could also help reduce the participant burden and encourage those who delayed starting or withdrew in traditionally busy seasons (eg, holidays), a trend also seen in other recruitment studies [36]. The conclusion survey also highlighted technological issues (app crashes, time discrepancies) that affected adherence to the study protocol. The feedback gathered from the conclusion survey will inform necessary technology fixes before this study design is implemented at a larger scale, including the potential to access the app on a personal device rather than an unfamiliar study phone. Lastly, participants reported internet disturbances (eg, power outages, loss of Wi-Fi) that hindered data submission, highlighting the risks associated with technology-based studies.

### Future Directions

Insights gained from this feasibility study can inform larger studies, ultimately paving the way for advanced deep learning approaches to identify ovulation in people with irregular menstrual cycles. Future studies must optimize participant completion by targeting recruitment to address frequent ineligibility, ensuring sufficient study materials, reducing travel burdens, enhancing study team support and communication, and incorporating health education to equip participants with ovulatory health information to ameliorate the potential stress impacts of observing anovulation. Because previous research indicates the cycle length varies by race and ethnicity, for improved AI model function in the worldwide population, future studies should recruit broadly from diverse race and ethnicities [39-43]. These optimization techniques can broadly be applicable to remote studies with high daily data demand.

The potential advanced deep learning approaches for ovulation prediction in people with irregular menstrual cycles has strong clinical implications. This advancement could help those with irregular cycles better understand their health. Additionally, it could serve as a potential fertility management strategy before engaging with a fertility clinic and a time-and resource-intensive process. Understanding which menstrual cycles are ovulatory by means of salivary ferning in this population could also help clinicians better understand the health of their patients.

### Conclusion

Robust AI models typically require large amounts of data for training, and an AI model designed for ovulation prediction would need data to learn from and to test its parameters effectively. A total of 7 participants with PCOS, some of whom experience irregular or unpredictable cycles, were able to complete daily, demanding tasks without in-person study team oversight, attend lab visits, and return biological samples to the study team. This feasibility study revealed limitations, which after optimization, when scaled, will provide an AI model with sufficient data to develop a smartphone-based ovulation tracker specifically tested on females with irregular or unpredictable cycles, including those with PCOS. The study’s insights can be generalized to broader female health and femtech research, especially as the medical sphere shifts into more remote spaces, and can be used to advance femtech in AI-centered areas.

## Appendix

Multimedia Appendix 1 Recruitment post on the MGH Rally website to promote the study. This was 1 of 2 methods used for participant recruitment. MGH: Massachusetts General Hospital.

Multimedia Appendix 2 Recruitment letter sent to targeted patients via the MGH Patient Gateway system. This was 1 of 2 methods used for participant recruitment. MGH: Massachusetts General Hospital.

Multimedia Appendix 3 Study instruction manual provided to eligible participants who received a study kit to explain the study tasks.

Multimedia Appendix 4 Study calendar provided to eligible participants who received a study kit to explain the cadence of their study tasks.

Multimedia Appendix 5 Demographic breakdown of participants who completed the study (n=7).

## Abbreviations

AI: artificial intelligence
BBT: basal body temperature
CNN: convolutional neural network
femtech: technology sector focused on enhancing female health
GBA: Greater Boston area
LH: luteinizing hormone
MGH: Massachusetts General Hospital
NaCl: sodium chloride
PCOS: polycystic ovary syndrome
PEONy: Precision Ovulation Prediction for Patients with PCOS
REDCap: Research Electronic Data Capture

## Data Availability

The datasets generated or analyzed during this study are available from the corresponding author upon reasonable request.
