# Supplementary material for: Digitally Enabled AI-Interpreted Salivary Ferning–Based Ovulation Prediction: Feasibility Study
Source: J Med Internet Res. 2025 Aug 5;27:e73028. doi: 10.2196/73028 (PMC12365558; doi:10.2196/73028)
Supplement: Multimedia Appendix 1 [file jmir_v27i1e73028_app1.pdf]

Mass General Brigham

[Date]

[Patient name
Address
City, State, Zip]

Dear [Ms. Patient],

We are writing to let you know about a research study that might interest you. Mass General Brigham is committed to providing excellent care to you and to our community. An important part of our mission is to learn new ways to care for our patients by doing research. Our patients play an important role in research by joining studies to help discover better treatments or better ways to prevent health problems.

Mass General Brigham is a health system that includes hospitals, community health centers, and groups of doctors. We work together to provide the best care possible to our community. Patients at Mass General Brigham have access to high-quality care across our entire system. As a healthcare system, we also work together to provide access to research studies to all our patients.

**The information below tells you about the study that might interest you. You can learn more or speak with the research team to decide if it is a good match for you.**

**Study Name: A personalized smartphone-based assay for at-home ovulation prediction in women including those with polycystic ovarian syndrome (PCOS) – The Peony Study**

**What we are studying:**

We are doing research to develop an at home ovulation predictor kit based on saliva. Most at-home ovulation tests are based on urinary luteinizing hormone (LH) and work well for women with regular predictable menstrual cycles. However, Women with polycystic ovarian syndrome (PCOS) are often unable to predict ovulation using urinary luteinizing hormone (LH)-based tests, the most common ovulation test due to tonically elevated LH levels and false positive results.

**Who might qualify:** Inclusion: Age 18 to 35 years old, a person who is currently menstruating with a cycle variation of +/- 4 days or a person who has been diagnosed with PCOS, ability to read and comprehend English, and weighs more than 110 pounds. They will need to also have an active PCP or Primary GYN provider.

History of surgical menopause (no ovaries or no uterus), history of or current chemotherapy or radiation, currently breastfeeding and/or post conception. Current use of hormonal therapy of any form for any purpose that would interfere with ovulation status. Participants who have stopped hormonal therapy are eligible upon having one menstrual cycle after

cessation of hormonal therapy. People with thyroid or prolactin disorders will be excluded.

These may or may not apply to you.

**What you are asked to do in the study:**

If you agree, you will be expected to do the following activities, take two surveys a total 10-15 minutes per survey, and then provide a saliva sample every day of the study for up to 2 menstrual cycles or 4 months. The sample collection will take about 15 minutes each day. You will be in the study for up to 4 months if you decide to stay for the whole study. You will be compensated up to 100 dollars for your participation. 50 dollars will be given to you once you complete your first cycle, and you will receive another 50 dollars once you complete your second cycle. You will be given 75 dollars if you choose to complete an optional conclusion survey once your participation in the study concludes. The conclusion survey will take approximately 10 minutes to complete.

**For more information:**

To learn more about <u>THIS</u> study: Contact [the study coordinator TBD at XX-XXX-XXXX. / Study Link]

After you learn more, you can decide whether to join or not. We may also find out that you do not qualify for the study. Whether you join the study or not, it will not change the medical care you receive here at Mass General Brigham.

To learn more about research in general:

Call the Research Navigator Office: **857-282-5370**

Go to the Rally website: rally.partners.org/research

If you do not want to receive notices about *any* studies at Mass General Brigham:

- Log in to your Patient Gateway account.
  On the Menu scroll down to “Resources” and choose “Research Opportunities”
  Read the brief information and chose the appropriate button.

***OR***

- Contact the Research Navigator Office: **Call 857-282-5370**

Mass General Brigham

Thank you in advance for considering this research study.

Sincerely,

Shruthi Mahalingaiah, MD, MS, FACOG
Pronouns: She, her, hers
Director of Clinical Research
Division of Reproductive Endocrinology and Infertility

**Massachusetts General Hospital | Ovulation Health Clinic**
55 Fruit St, Yawkey 4, Boston, MA 02114
T 617-724-6850 | F 617-724-3498
smahalingaiah@partners.org
massgeneralbrigham.org
