# Supplementary material for: Digitally Enabled AI-Interpreted Salivary Ferning–Based Ovulation Prediction: Feasibility Study
Source: J Med Internet Res. 2025 Aug 5;27:e73028. doi: 10.2196/73028 (PMC12365558; doi:10.2196/73028)
Supplement: Multimedia Appendix 2 [file jmir_v27i1e73028_app2.pdf]

Mass General Brigham

This project is in preview mode.

The Peony Study

This study aims to develop at-home fertility testing targeted towards women with PCOS or irregular cycles done through the use of saliva ferning.

4 months
ESTIMATED TIME COMMITMENT

Female, Transgender Male, Nonbinary, Other, Gender-expansive, 18-35 years
MAY BE ELIGIBLE

Payment up to $100
MAY BE OFFERED

Survey, Blood draw
MAY BE REQUIRED

- Massachusetts General Hospital
- Polycystic Ovary Syndrome

This project is not recruiting.

Overview

What we are studying

At-home ovulation predictor kits empower women with valuable information on their ovulatory status and fertility, allowing them to make informed decisions. Most at-home ovulation tests are based on urinary luteinizing hormone (LH) and work well for women with regular predictable menstrual cycles. However, Women with polycystic ovarian syndrome (PCOS) are often unable to predict ovulation using urinary luteinizing hormone (LH)-based tests, the most common ovulation test due to tonically elevated LH levels and false positive results. PCOS affects over 15% of women within the reproductive age, leading to abnormal hormone profiles, disordered ovulation, and irregular menstrual cycles and is a leading contributor to ovulatory infertility.

Share

Principal Investigator

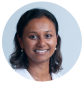

Shruthi Mahalingaiah
Massachusetts General Hospital

Without a reliable at-home test for ovulation prediction, women with PCOS are more limited in their ability to plan and make important life choices around fertility and health. Furthermore, complicating the understanding of ovulation in women with PCOS, is that everyone has their own cycle features. This results in the need for women with PCOS to seek medical intervention for early support in attempting conception, which can be limiting to women who are under-insured or are in states without any mandated coverage for fertility services. Furthermore, the risk of endometrial hyperplasia and cancer increases in women with PCOS, with risk elevations in women with fewer than 4 ovulatory periods per year for 4 years or more and is further accelerated in the setting of obesity and diabetes. Understanding and tracking the ovulatory cycles per year with an easy-to-use, affordable, reliable, and personalized at-home test that does not require LH to determine ovulatory status may allow not only ovulation prediction for fertility but also help risk stratify and intervene to support patients and reduce

the risk of endometrial cancer. Currently, there is no technology or product available to women with PCOS to reliably identify or track their ovulation cycles at-home. Building upon our extensive experience in the development of mobile health diagnostic systems and ovulation/infertility we propose a smartphone-based salivary ferning test, empowered by artificial intelligence, for ovulation detection and tracking for women, inclusive of women with PCOS (Fig. 1, All Figures in Appendix A). In contrast to traditional hormone-mediated tests that rely on LH, salivary ferning-based ovulation prediction is linked to changes in the estrogen hormone levels.

Changes in estrogen concentration led to changes in the concentrations of salivary electrolytes, particularly sodium chloride (NaCl), and proteins. The interactions between sodium chloride and proteins in saliva generate fern-like structures due to the crystallization of NaCl and can be observed under a smartphone-based optical sensor. The concentration of estrogen, NaCl, and proteins

reaches its highest around 4 days before ovulation. Therefore, the fern patterns are at their maximum density at this time frame and up to the day of ovulation. Additionally, the unique ferning response linked with everyone's hormone profile can be capitalized upon by our artificial intelligence algorithm for accurate and personalized predictions.

Who can participate

Inclusion: Age 18 to 35 years old, a person who is currently menstruating with a cycle variation of +/- 4 days or a person who has been diagnosed with PCOS, ability to read and comprehend English, and weighs more than 110 pounds. They will need to also have an active PCP or Primary GYN provider.

Who cannot participate

Exclusion: History of surgical menopause (no ovaries or no uterus), history of or current chemotherapy or radiation, currently breastfeeding and/or post

conception. Current use of hormonal therapy of any form for any purpose that would interfere with ovulation status. Participants who have stopped hormonal therapy are eligible upon having one menstrual cycle after cessation of hormonal therapy.

What you may be asked to do

Participants will be asked to do the following:

Take Surveys

Provide Saliva Samples

Provide Urine Smaples

Provide Blood Samples

Project activities may include:

- Survey
- Blood draw

Estimated Time Commitment

4 months

What You May Get

Up to $100 of compensation for the study.

Location

Mass General Hospital
55 Fruit St, Boston, MA 02114

Map it!

Travel

- Parking reimbursed

- Accessible by public transportation

Travel and Parking Details

Additional Information

Participating Institutions

- Massachusetts General Hospital

Funding Source

- Department

About Us

What is Rally?

Share your story

Terms of use

Privacy policy

Learn

Research at Mass General Brigham

Research Education

For Researchers

Submit new project

Manage projects

Resource center

Upcoming trainings

Need help?

Using Rally

Frequently Asked Questions

Contact us

Sign up to our weekly email to get the latest research opportunities.

Enter Your Email Address

Submit

© 2024 Mass General Brigham Incorporated.
All Rights Reserved.

Rally v2.2.3.1-RELEASE
