# Supplementary material for: Digitally Enabled AI-Interpreted Salivary Ferning–Based Ovulation Prediction: Feasibility Study
Source: J Med Internet Res. 2025 Aug 5;27:e73028. doi: 10.2196/73028 (PMC12365558; doi:10.2196/73028)
Supplement: Multimedia Appendix 3 [file jmir_v27i1e73028_app3.pdf]

PEONY STUDY

ONBOARDING MANUAL

Principal Investigator

SHRUTHI MAHALINGAIAH, MD, MS, FACOG

STUDY KIT CONTENTS

MATERIALS FOR DATA COLLECTION

Smartphone

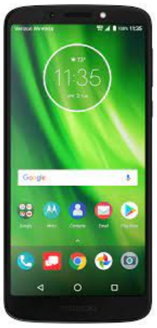

The PEONY Study App

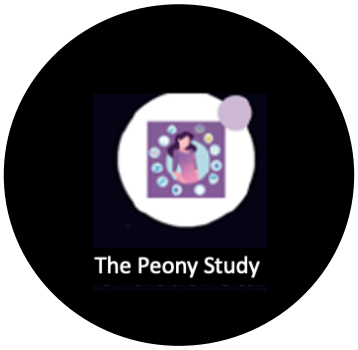

MATERIALS FOR SALIVA COLLECTION

Optical Attachment

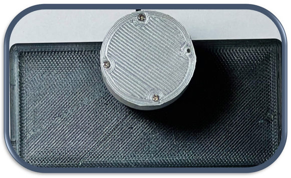

Front

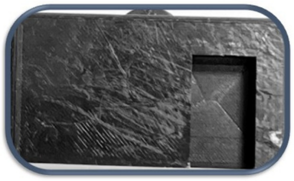

Back

Sample Slide

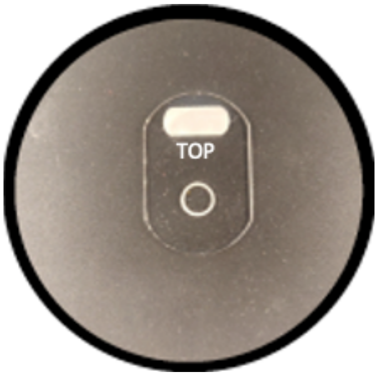

MATERIALS FOR OVULATION PREDICTION (LH) COLLECTION

Ovulation Predictor Kit

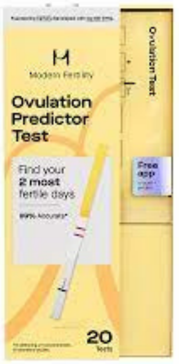

Urine Cup for Ovulation Kit

SAMPLE COLLECTION PREPARATION

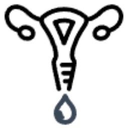

Start saliva collection on the first day of your period

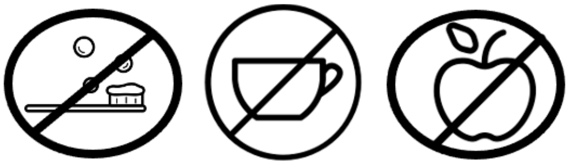

Do not brush your teeth, ear, or drink prior to collecting saliva each day

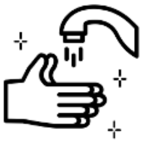

Always wash and dry your hands thoroughly before you start any data collection process.

TEST PROCEDURE, CYCLE 1

CYCLE 1, DAY 1 - FIRST DAY OF YOUR PERIOD

1

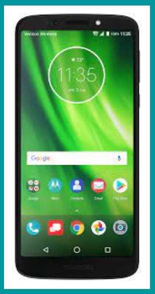

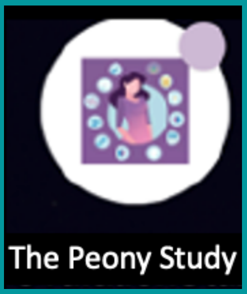

Open the PEONY app on the smartphone. Log-in with the study information provided by the study team.

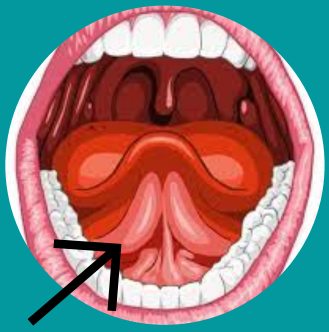

[1]

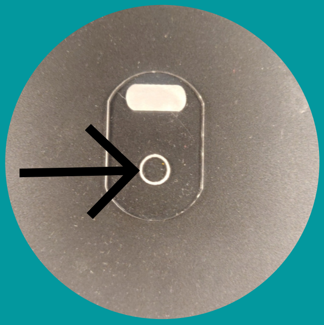

[2]

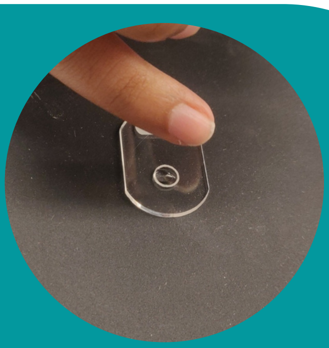

[3]

2

Take a saliva sample from under the tongue using a finger [1] & dab a drop onto the circle etched [2] into the slide as shown in [3].

3

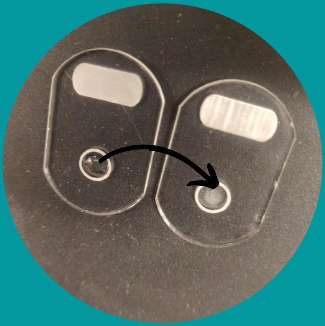

[4]

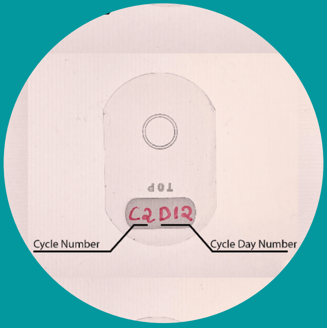

[5]

Wait for the sample to dry (10-15mins) at room temperature [4]. Write your cycle number and cycle day on the slide in the format "C # D #" as shown [5].

4

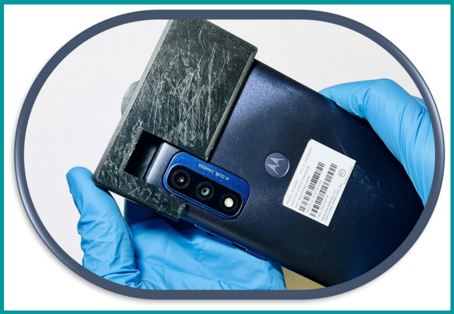

[6]

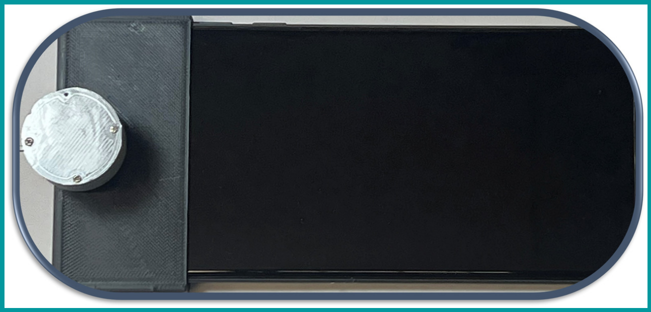

[7]

Insert the mobile phone into the optical device slot as demonstrated in the picture [6], after which the anterior of the assembly should resemble the picture [7].

5

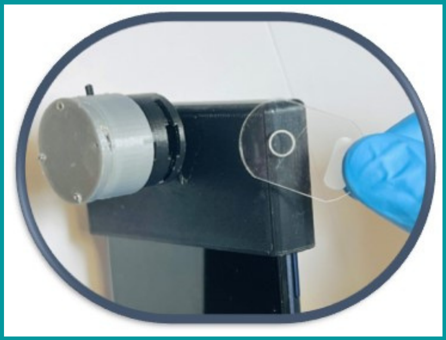

[8]

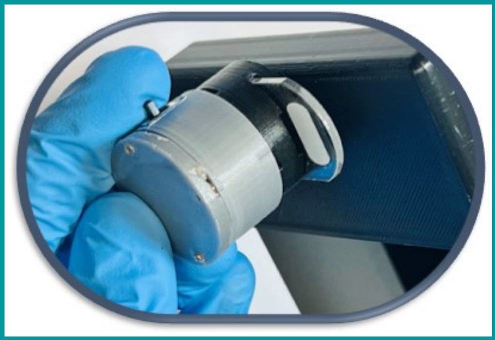

[9]

Insert the slide into the optical equipment's slot with the etched part facing upwards as shown in picture [8] and switch on the LED as shown in picture [9]. Make sure the dry sample is in focus and within the camera area.

Click on the pink camera in the bottom right corner of the screen. Allow the app to take photos and capture the saliva image. Place the used saliva chip into the labeled. container

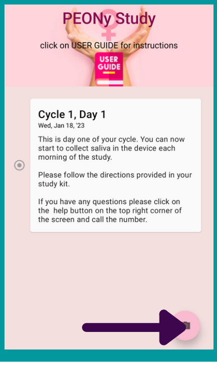

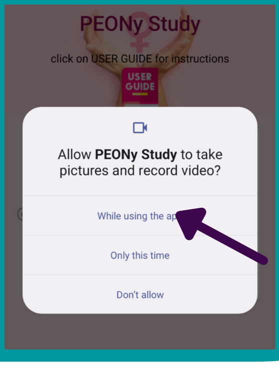

Cycle 1, Days 2-9

Repeat the test procedure (Steps 1-6) performed on Cycle 1, Day 1.

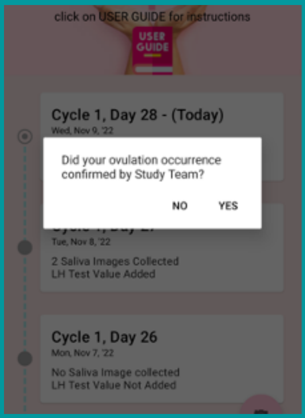

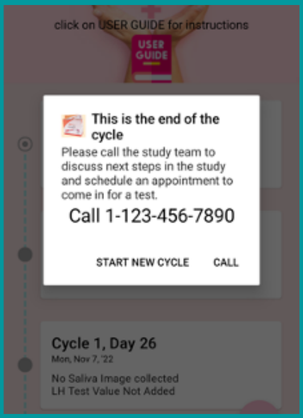

Cycle 1, Day 10

Repeat the test procedure performed on Cycle 1, Days 1-9. Then, perform the Modern Fertility ovulation prediction test using the box and urine cup provided in the study kit. Please collect the urine sample according to the instructions Modern Fertility provides in the box. Enter the LH Test Value obtained from the ovulation prediction test in the PEONY Study app. Overall, for Cycle 1, Day 10, you should have both a saliva sample image uploaded **AND** the LH Test Value obtained from the ovulation prediction test entered into the PEONY Study app.

NOTE: Please contact the study team immediately if you miss a day of sample collection. You may need to restart data collection.

If positive ovulation is confirmed by the study team, select "Yes" when the app asks "Was your ovulation occurence confirmed by Study Team?" The app will prompt you to call the study team to schedule a lab visit at Massachusetts General Hospital (MGH) to collect a blood and urine sample within 7-10 days of your at-home positive test.

NOTE: If the result from your LH test is not positive on Day 10, please continue repeating the test procedure, collecting a urine sample, **AND** entering an LH Test Value into the study app each day until you get a positive ovulation result.

After Cycle 1, Day 10

Continue repeating the test procedure for each day of your cycle up until the day of your lab visit at MGH. The day after your lab visit, continue the test procedure for each day of your cycle until you are contacted by the study team about your lab results.

Once the study team has verified that your confirmatory lab test at MGH is positive, stop collecting saliva samples until your next period.

TEST PROCEDURE, CYCLE 2

CYCLE 2, DAY 1 - FIRST DAY OF YOUR NEXT PERIOD

On the first day of your next period, repeat the test procedure (Steps 1-6) performed on Cycle 1, Day 1.

CYCLE 2, DAYS 2-9

Repeat the test procedure performed on Cycle 2, Day 1.

CYCLE 2, DAY 10

Repeat the test procedure performed on Cycle 1, Days 1-9. Then, perform the Modern Fertility ovulation prediction test using the box and urine cup provided in the study kit. Enter the LH Test Value obtained from the ovulation prediction test in the PEONY Study app. For Cycle 2, Day 10, you should have both a saliva sample image uploaded **AND** the LH Test Value obtained from the ovulation prediction test entered into the PEONY Study app.

If positive ovulation is confirmed by the study team, select "Yes" when the app asks "Was your ovulation occurence confirmed by Study Team?" The app will prompt you to call the study team to schedule a lab visit at Massachusetts General Hospital (MGH) to collect a blood and urine sample within 7-10 days of your at-home positive test.

AFTER CYCLE 2, DAY 10

Once again, continue repeating the test procedure for each day of your cycle up until the day of your lab visit at MGH.
