# Supplementary material for: Digitally Enabled AI-Interpreted Salivary Ferning–Based Ovulation Prediction: Feasibility Study
Source: J Med Internet Res. 2025 Aug 5;27:e73028. doi: 10.2196/73028 (PMC12365558; doi:10.2196/73028)
Supplement: Multimedia Appendix 4 [file jmir_v27i1e73028_app4.pdf]

STUDY CALENDAR

Please use this study calendar to keep track of the messages you receive on the Peony app and the actions you should take as you progress through the study!

Day of Cycle	Trigger	Message	Participant Action
NA	When the participant opens the app for the first time	Welcome to the app and study. Please note that you should start the study on your first bleed day of your menstrual cycle. Once that occurs you can start saliva collection	Participant waits to start study until their first bleed day
Bleed day 1/Cycle Day 1	When the participant logs their first bleed day	This is day one of your cycle. You can now start to collect saliva in the device each morning of the study. Please follow the directions provided in your study kit. If you have any questions please call [RA Name and Phone Number]	Participant provides their first saliva sample
Cycle day 2 – Cycle Day 9	Study participation	Please remember to collect your saliva first thing in the morning before you eat, drink or brush your teeth.	Participant provides saliva sample
Cycle Day 9	When the participant should collect their first LH sample on cycle day 10	Tomorrow is day 10 of your cycle. Ovulation can be triggered by the body's leutenizing hormone (LH) surge. While not everyone has a predictable surge, we would like you to test for it to help understand your body's functioning better. Tomorrow is the first day you should collect your modern fertility sample. Please collect the sample according to the instructions Modern Fertility provides in the box.	Participant is prepared to provide LH sample
Cycle day 10 – Until ovulation positive occurs	Study participation	Good morning, Please remember to provide both a saliva sample and urine sample this morning.	Participant provides samples
Cycle day 10 - Cycle Day 35	Positive Ovulations Event	You have tested positive for ovulation. Please call the study office to set up an appointment to provide a blood sample and a urine sample. Call [number] or email [study email]. If you call and we are not available, please leave a message with the best number and time to call you back.	Participant calls study office.
After positive ovulation event – Until cycle day 1	Study participation	Please remember to collect your saliva first thing in the morning before you eat, drink or brush your teeth.	Participant provides samples
Cycle Day 38	No positive ovulatory event	Please call the study team to discuss next steps in the study and schedule an appointment to come in for a test.	Participant calls the study team to set up an Anovulatory lab.
Cycle 2 Prompt after they come in for a lab	Lab test occurred	Remember to log your Period Day 1 to start your next study cycle tracking.	
