# Supplementary material for: Digitally Enabled AI-Interpreted Salivary Ferning–Based Ovulation Prediction: Feasibility Study
Source: J Med Internet Res. 2025 Aug 5;27:e73028. doi: 10.2196/73028 (PMC12365558; doi:10.2196/73028)
Supplement: Multimedia Appendix 5 [file jmir_v27i1e73028_app5.docx]

This is a Multimedia Appendix to a full manuscript published in the J Med Internet Res. For full copyright and citation information see <http://dx.doi.org/10.2196/jmir.xxxx>

Demographic breakdown of participants that completed the study (n=7).

| Participant | Race/Ethnicity | Age | Body Mass Index (kg/m^2^) | Menstrual Cycle Length (days) | PCOS Status |
| --- | --- | --- | --- | --- | --- |
| 1 | White | 33 | 31.80 | 30-31 | Professional Diagnosis |
| 2 | White | 31 | 23.90 | 36-39 | Professional Diagnosis |
| 3 | Brazilian | 33 | 39.70 | 26-29 | Professional Diagnosis |
| 4 | Hispanic | 34 | 25.05 | 32-35 | Professional Diagnosis |
| 5 | Hispanic | 19 | 35.92 | 40+ | Professional Diagnosis |
| 6 | White | 35 | 33.91 | 30-31 | Professional Diagnosis |
| 7 | Middle Eastern | 22 | 34.86 | 30-31 | Professional Diagnosis |
